# Synergistic action of phage phiIPLA-RODI and lytic protein CHAPSH3b: a combination strategy to target *Staphylococcus aureus* biofilms

**DOI:** 10.1038/s41522-021-00208-5

**Published:** 2021-04-22

**Authors:** Ana Catarina Duarte, Lucía Fernández, Vincent De Maesschalck, Diana Gutiérrez, Ana Belén Campelo, Yves Briers, Rob Lavigne, Ana Rodríguez, Pilar García

**Affiliations:** 1 Instituto de Productos Lácteos de Asturias (IPLA-CSIC), Paseo Río Linares s/n 33300, Villaviciosa, Asturias Spain; 2 DairySafe Group. Instituto de Investigación Sanitaria del Principado de Asturias (ISPA), Oviedo, Spain; 3 Laboratory of Applied Biotechnology, Department of Biotechnology, Faculty of Bioscience Engineering, Ghent University, Ghent, Belgium; 4 Laboratory of Gene Technology, Department of Biosystems, Faculty of Bioscience Engineering KU Leuven, Heverlee, Belgium

**Keywords:** Antimicrobials, Biofilms, Pathogens

## Abstract

*Staphylococcus aureus* is considered a priority pathogen due to its increasing acquisition of antibiotic resistance determinants. Additionally, this microbe has the ability to form recalcitrant biofilms on different biotic and inert surfaces. In this context, bacteriophages and their derived lytic proteins may be a forward-looking strategy to help combat staphylococcal biofilms. However, these antimicrobials exhibit individual limitations that may be overcome by combining them with other compounds. This work investigates the combination of a phage-derived lytic protein, CHAPSH3b, and the virulent bacteriophage phiIPLA-RODI. The obtained results show the synergy between both antimicrobials for the treatment of 24-h-old *S. aureus* biofilms, with greater reductions in viable cell counts observed when phage and lysin are applied together compared to the individual treatments. Time-kill curves and confocal microscopy revealed that the fast antibacterial action of CHAPSH3b reduces the population up to 7 hours after initial exposure, which is subsequently followed by phage predation, limiting regrowth of the bacterial population. Moreover, at least 90% of bacteriophage insensitive mutants are susceptible to the lytic protein. Therefore, CHAPSH3b might help curtail the development of phage resistance during treatment. The combination of the lysin and phiIPLA-RODI also showed promising results in an ex vivo pig skin model of wound infection. Overall, the results of this study demonstrate that the combination of phage-derived lytic proteins and bacteriophages can be a viable strategy to develop improved antibiofilm products.

## Introduction

*Staphylococcus aureus* is a Gram-positive human opportunistic pathogen responsible for multiple infections^1,2^, including food poisoning due to the secretion of heat-stable enterotoxins^3,4^. Indeed, this microbe is armed with an arsenal of virulence factors, including numerous toxins, immune evasion factors, and molecules involved in biofilm development^3^.

Biofilm formation is a complex process involving the initial adherence of bacterial cells to a surface, followed by the production of an extracellular matrix^5–7^. Importantly, biofilm-embedded cells are known for their increased ability to withstand antibiotics and disinfectants compared to planktonic cells^8^. This makes biofilms the perfect reservoir for pathogenic bacteria on surfaces of clinical and industrial settings. As such, biofilm formation by this pathogen simultaneously favors persistent infection, antibiotic resistance, and immune evasion^9^. Moreover, biofilms are considered to be involved in at least 65% of all infections in humans^10,11^. In *S. aureus*, one of the major matrix components is polysaccharide intercellular adhesin (PIA)/poly-N-acetyl-1,6-b-glucosamine (PNAG), which is synthesized by the proteins encoded by the intercellular adhesion (*ica*) operon^12^ and provides structural integrity to the biofilms. However, some surface proteins, such as protein A^13^ or the biofilm-associated protein (Bap)^14,15^, as well as extracellular DNA (eDNA) also contribute to biofilm matrix development and stabilization^5^.

Antimicrobial resistance has become a major medical threat worldwide and, in this context, *S. aureus* is currently considered a priority pathogen. For example, the World Health Organization (WHO) has estimated that 60% of all reported *S. aureus* infections in Europe are caused by methicillin-resistant strains (MRSA)^16^. Also, resistance to vancomycin, the antibiotic of choice to treat MRSA infections, can be a cause of concern^17^. More recently, the lipopeptide daptomycin was introduced for the treatment of complicated staphylococcal infections, but resistant strains have also been isolated since then^18^.

In this scenario, bacteriophages (phages) and their derived proteins have been proposed as an alternative or complementary strategy to conventional therapeutics that may help to control the spread of antibiotic resistance in bacterial pathogens. One of the advantages of bacteriophages is their specificity against one bacterial genus or species, being innocuous against non-target bacteria. Moreover, phages are the most abundant biological entities on earth, multiply themselves naturally, and are safe for humans^19,20^. Typically, phages degrade the structural peptidoglycan present in the bacterial cell wall using two classes of lytic proteins: virion-associated peptidoglycan hydrolases (VAPGHs) degrade peptidoglycan in the initial steps of the infection, and endolysins help release the phage progeny during the late phase of the lytic cycle^21–24^. The modular structure of lytic proteins facilitates the design of new chimeric proteins via domain shuffling, which frequently displays improved lytic activity^22,25,26^ and overall enhanced traits^27^. These enzymes can be used as antibacterial agents targeting bacteria from the outside, accessing the peptidoglycan, and destroying the cell walls^28–30^. Furthermore, the rate of selection of bacterial resistance to lysins is very low^31^ and, while still being quite specific, their spectrum of action generally exceeds that of bacteriophages. For all these reasons, phage lytic proteins constitute promising antimicrobial candidates.

Nevertheless, there are some disadvantages associated with the therapeutic use of both phages and phage-derived proteins. In the case of phages, it is common to observe the selection of bacteriophage insensitive mutants (BIMs) during therapy^32^, together with their narrow host range^33^, and their potential contribution to horizontal gene transfer^34^. Similarly, there are also concerns associated with the use of phage lytic proteins. For instance, unlike phages, the concentration of these proteins decreases gradually after administration, as is also the case for standard-of-care antibiotics. Furthermore, it is important to ensure protein stability under the desired environmental conditions to avoid protein inactivation by factors like pH, temperature, or degradation by proteases amongst others^35^.

One way to overcome the individual shortcomings of bacteriophages or lysins is by combining them with other antimicrobial agents. Indeed, several studies have demonstrated that the combination of phages with antibiotics or antiseptics is promising, exhibiting a synergistic effect in biofilm removal experiments^36–38^. Another strategy is the combination of multiple phages targeting different receptors in a single phage preparation, known as a phage cocktail^39,40^. Phage lytic enzymes have also been combined with other antimicrobials like antibiotics^41–43^ or used as part of a multi-enzyme approach by mixing them with depolymerases, which target polysaccharides such as those present in the extracellular matrix of biofilms^44^. However, to our knowledge, no study has found synergistic effects between phages and lytic proteins.

In our previous work, we showed that chimeric protein CHAPSH3b, which consists of the CHAP domain from peptidoglycan hydrolase HydH5 and the SH3b cell wall binding domain (CBD) from lysostaphin^45^, displays antistaphylococcal activity in growth medium and milk, as well as biofilm-removal properties^45,46^. Furthermore, CHAPSH3b inhibits *S. aureus* biofilm formation, presumably by the downregulation of autolysin-encoding genes^46^. Moreover, we have characterized the virulent phage vB_SauM_phiIPLA-RODI (phiIPLA-RODI), which is also effective in eliminating staphylococcal biofilms^39^. This study aimed to assess the potential interactions between phage phiIPLA-RODI and the phage-derived chimeric lytic protein CHAPSH3b when used together for biofilm removal.

## Results

### Phage phiIPLA-RODI and chimeric lysin CHAPSH3b act synergistically to remove staphylococcal biofilms

To examine the potential interactions between phage phiIPLA-RODI and the lytic protein CHAPSH3b for biofilm removal, three *S. aureus* strains were chosen on the basis of their biofilm formation ability and matrix composition. These strains include *S. aureus* V329 and 15981, which have a strong biofilm production phenotype, and *S. aureus* IPLA1, a weak biofilm producer^15,47^. Regarding their matrix composition, *S. aureus* V329 biofilm is mostly composed of Bap (biofilm-associated protein) and eDNA, whereas both *S. aureus* 15981 and IPLA1 biofilms mainly consist of exopolysaccharides^47^.

First, the susceptibility of the three strains to phiIPLA-RODI and CHAPSH3b was determined by performing minimum inhibitory concentration (MIC) assays. The MIC values of the chimeric protein were quite similar for all three strains. Indeed, *S. aureus* IPLA1 and V329 strains showed identical susceptibility with a MIC of 60.5 µg/ml (~2 µM), whereas strain *S. aureus* 15981 had a higher MIC of 121.05 µg/ml (~4 µM). For phage phiIPLA-RODI, strain *S. aureus* IPLA1 was the most susceptible, with a MIC of 10^3^ PFU/ml, followed by *S. aureus* V329 with a MIC of 10^9^ PFU/ml and, finally, *S. aureus* 15981, with a MIC over 10^9^ PFU/ml. In addition, the efficiency of plating (EOP) of phiIPLA-RODI on strains IPLA1, 15981, and V329 were 1.0 ± 0.00, 0.80 ± 0.02, and 0.55 ± 0.19, respectively. In turn, the adsorption rates on strains IPLA1, 15981, and V329 were not significantly different from each other (83.77 ± 2.41, 85.34 ± 14.37, and 79.97 ± 6.53%, respectively).

**Fig. 1:**
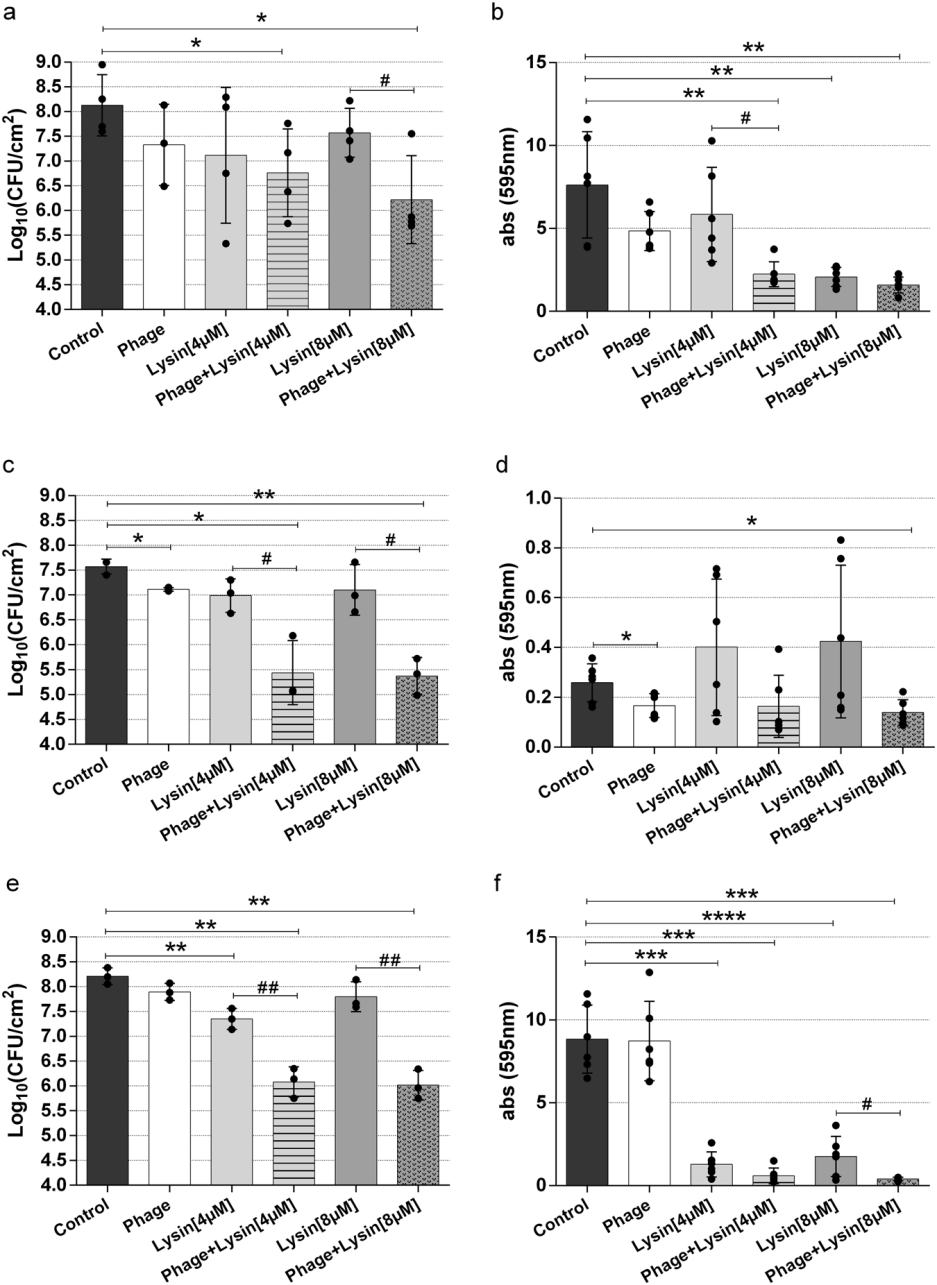
Treatment of preformed biofilms formed by different *S. aureus* strains. Biofilms formed by *S. aureus* 15981 (**a** and **b**), IPLA1 (**c** and **d**), and V329 (**e** and **f**) were treated with bacteriophage phiIPLA-RODI (10^10^ PFU/ml) (white bars), chimeric protein CHAPSH3b (4 and 8 µM) (light gray and dark gray bars, respectively) or the combination of both antimicrobials (striped and dotted bars). Biofilms were allowed to develop for 24 h and then treated for another 24 h at 37 °C. TSB medium alone was added to the control wells (black bars). After incubation, the viable cell counts of the three strains were determined (**a**, **c** and **e**) and the adhered biomass was quantified (A_595_) by crystal violet staining (**b**, **d**, and **f**). Data represent the means ± standard deviations of three independent experiments. Bars with an asterisk are statistically different from the untreated control, and bars with a hash sign are statistically different from the treatment with CHAPSH3b alone at the same concentration, according to the unpaired *t*-test with Welch’s correction. *^/#^*p*-value < 0.05, **^/##^*p*-value < 0.01, ***^/###^*p*-value < 0.001 and ****^/####^*p*-value <0.0001.

Next, to establish the antibiofilm potential of these phage-derived antimicrobials, 24-h biofilms of the three strains were treated with different combinations of the phage at 10^10^ PFU/ml and the chimeric protein at 4 or 8 µM. The results indicated a potential synergy between phiIPLA-RODI and CHAPSH3b (Fig. 1). Treatment of biofilms formed by *S. aureus* 15981 with the phage or the protein alone did not significantly affect the number of viable cells compared to the untreated control (Fig. 1a). Conversely, the combination of both antimicrobials did lead to a significant reduction in viable cells of 1.4 (*p* = 0.0498) and 1.9 log units (*p* = 0.0152) at protein concentrations of 4 and 8 µM, respectively (Fig. 1a). These results suggested a synergistic effect between phage and protein at 8 µM with an interaction index of 0.55. Interestingly, the interaction between the phage and the protein at 4 µM was additive with an interaction index of −0.45. Regarding total biomass, the addition of the phage alone or the protein at 4 µM did not have any significant impact, whereas treatment with CHAPSH3b at 8 µM resulted in a significant biomass reduction (*p* = 0.0076) (Fig. 1b). The combination treatment always led to a decrease in total attached biomass (Fig. 1b). In *S. aureus* IPLA1 biofilms, there was a significant reduction in viable cells when applying the bacteriophage individually (0.5 log units) (*p* = 0.0284) or in combination with both concentrations of the protein (2.1 (*p* = 0.0239) and 2.2 log units (*p* = 0.0039) corresponding to 4 and 8 µM of CHAPSH3b, respectively), but not with CHAPSH3b alone (Fig. 1c). Thus, the combination of the phage with the chimeric protein at 4 and 8 µM had a synergistic effect with interaction index values of 1.10 and 1.27, respectively. Moreover, a significant reduction in total biomass was observed when using the phage alone (*p* = 0.035) or combined with 8 µM CHAPSH3b (*p* = 0.011) (Fig. 1d). Finally, in the case of strain *S. aureus* V329, a significant reduction in both viable cell counts (Fig. 1e) and biomass (Fig. 1f) was observed when combining the phage with the protein at different concentrations. Thus, a reduction of 0.9 log units (*p* = 0.0059) was observed when the biofilm was treated with the protein alone (4 µM), whereas a combination of the phage and the protein at 4 and 8 µM led to decreases of 2.1 (*p* = 0.0016) and 2.2 (*p* = 0.0013) log units, respectively. These results indicate that there was a synergistic effect in both cases, with interaction index values of 0.95 and 1.46 for 4 and 8 µM CHAPSH3b, respectively. Regarding total biomass, the protein alone or in combination with the phage significantly reduced the biofilm (*p*-values between < 0.0001 and 0.0002). Of note, the reduction in total biomass was higher in V329 than in the other two strains tested.

**Fig. 2:**
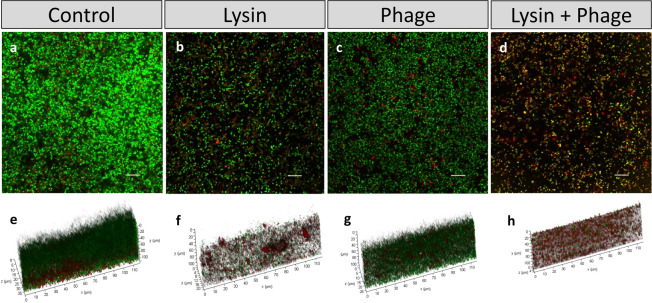
CLSM images of LIVE/DEAD-stained *S. aureus* V329 24-h-old biofilms after different treatments. Preformed *S. aureus* V329 biofilms were treated for 24 h at 37 °C with TSB alone (**a** and **e**), 8 µM CHAPSH3b (**b** and **f**), 10^9^ PFU/ml of phage phiIPLA-RODI (**c** and **g**) or a combination of 8 µM CHAPSH3b and 10^9^ PFU/ml of phage phiIPLA-RODI (**d** and **h**). Green cells were intact cells, whereas eDNA and cells with compromised cell-envelope integrity were stained in red. Scale bars represent 10 µm.

Based on these results, the *S. aureus* strain V329 and a protein concentration of 8 µM were selected for further experiments to examine these synergistic interactions more closely, since these conditions exhibited the highest interaction index. To better understand the effect of the combination treatment on biofilm structure, 24-h-old biofilms were treated with only protein or combined with the phage and compared to an untreated control by visualization with confocal microscopy (CLSM). After 24 h of incubation without treatment, strain *S. aureus* V329 displayed thick, well-structured biofilms (Fig. 2a and e). However, biofilm thickness was notably reduced after treatment with the protein alone (Fig. 2b and f). In contrast, treatment with the phage alone did not lead to any major change compared to the untreated control (Fig. 2c and g). However, the combination treatment had an even larger and more extensive impact than the protein alone (Fig. 2d and h). In addition, the latter biofilm contained a higher number of dead or compromised cells, which appeared red due to staining with propidium iodide (Fig. 2d and h).

### Phage predation curtails regrowth of the microbial population after CHAPSH3b inactivation

**Fig. 3:**
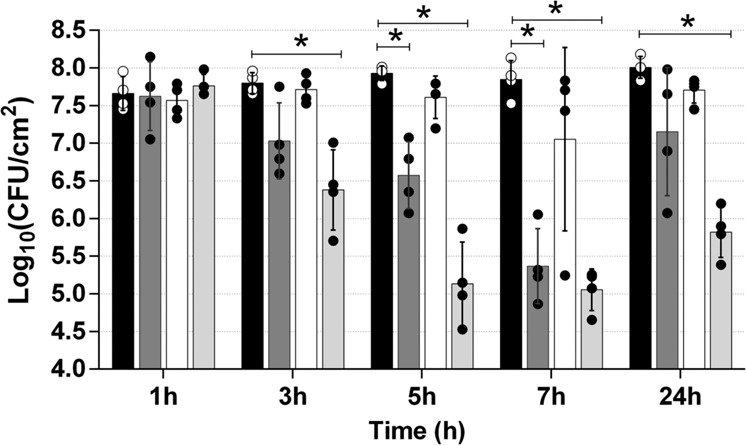
Time-kill curve of protein CHAPSH3b and/or phage phiIPLA-RODI against *S. aureus* V329 biofilms. 24-h-old biofilms were treated with protein at 8 µM (gray bars), phage at 1 × 10^9^ PFU/ml (white bars), or a combination of both (light gray bars) and incubated for 1, 3, 5, 7, or 24 h at 37 °C. Control wells were treated with TSB medium alone (black bars). Data correspond to the means ± standard deviations of four independent experiments and represented in logarithmic scale in colony-forming units per cm^2^ of biofilm. Bars with an asterisk are statistically different (*p* < 0.05) from the untreated control according to the Student’s t-test using the Holm-Sidak method.

**Fig. 4:**
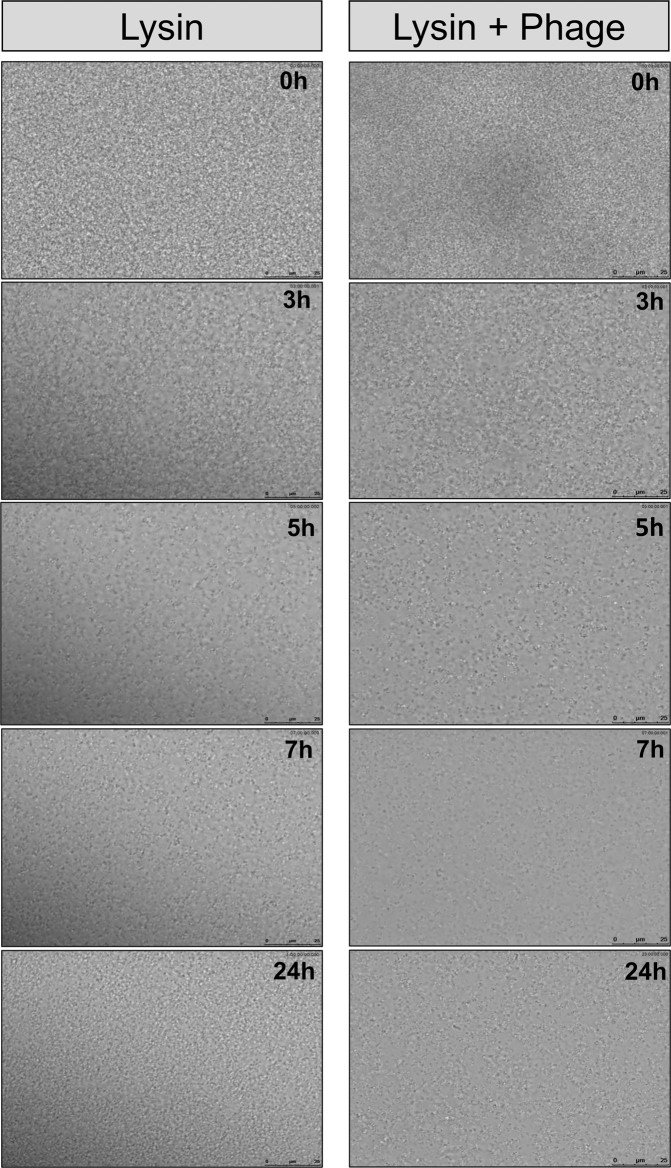
Time-lapse microscopy of S. aureus V329 biofilms subjected to different treatments. Time-lapse microscopy of 24-h-old *S. aureus* V329 biofilms treated with CHAPSH3b (8 µM) (left) or CHAPSH3b (8 µM) + phage phiIPLA-RODI (10^9^ PFU/ml) (right) during 24 h at 37 °C.

To study the killing dynamics of the combination therapy using phage phiIPLA-RODI and the chimeric protein CHAPSH3b, 24-h-old biofilms of strain *S. aureus* V329 were treated with a combination of phage (10^9^ PFU/ml) and protein (8 µM) or the two antimicrobials independently. CHAPSH3b exhibited a notable disrupting activity against the biofilm after just 3 h of treatment, judging by the reduction (0.7 log units) in attached bacterial counts (Fig. 3). Viable cell counts further decreased after 5 h of treatment (1.4 log units), and the maximum reduction (2.5 log units) was achieved after 7 h (Fig. 3). However, regrowth of the bacterial population was observed after 24 h of incubation at 37 °C, reaching viable cell counts similar to those of the untreated control. In contrast to the lytic protein, phage phiIPLA-RODI was not efficient in killing the bacterial cells attached to the surface at any time point. In spite of this, the phage did have an impact when combined with CHAPSH3b, demonstrating to be even more effective for biofilm removal than the protein alone. Thus, reductions in viable cell counts of 1.4 log units, 2.8 log units, and 2.8 log units were, respectively, observed after 3 h, 5 h, and 7 h of treatment. However, the most remarkable difference with the CHAPSH3b treatment was noted at the 24-h time point. In fact, even though the number of cells increased between the 7 h and 24 h time points in samples treated with the phage and protein combination, viable counts remained much lower than those in the untreated control (reduction of 2.2 log units). These results were subsequently confirmed by time-lapse microscopy analysis, although the action of the lysin stopped at an earlier time point under these conditions (Fig. 4) (See Supplementary Videos 1 and 2). Thus, when the biofilm was treated with CHAPSH3b alone, there was a gradual reduction in the number of viable cells up to 5 h post-treatment (Supplementary Video 1). However, after this time point, there is a gradual increase in bacterial cell coverage during the remaining incubation time. The results observed during the initial 5 h of incubation were fairly similar when treatment was carried out using a combination of the protein and phage (Supplementary Video 2). By contrast, cell proliferation after this time point was significantly slowed down by the presence of the phage. Indeed, the number of cells after 24 h of treatment was clearly reduced compared to the individual treatment with CHAPSH3b.

### Bacterial killing by CHAPSH3b increases the MOI of phiIPLA-RODI after three hours of treatment

**Fig. 5:**
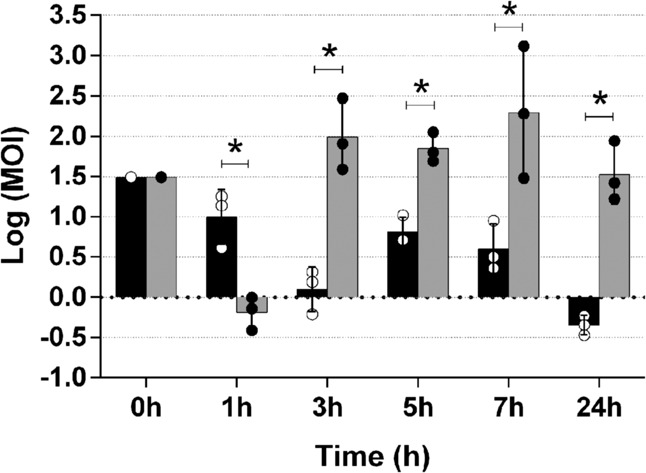
Changes in the MOI during incubation of *S. aureus* V329 biofilms treated with phage phiIPLA-RODI or a combination of phage and protein CHAP-SH3b. 24-h-old biofilms were treated with phage at 1 × 10^9^ PFU/ml (black bars) or a combination of phage at 1 × 10^9^ PFU/ml and protein at 8 µM (gray bars) and incubated for 1, 3, 5, 7, or 24 h at 37 °C. Data correspond to the means ± standard deviations of three independent experiments and represent the logarithm of the MOI for each time point. Bars with an asterisk are statistically different (*p* < 0.05) from each other according to the Student’s t-test using the Holm-Sidak method.

Next, we sought to explore the mechanism(s) of the synergy between phage phiIPLA-RODI and CHAPSH3b. One potential explanation for this phenomenon is that clearance of the biofilm by the protein might make bacterial cells more accessible to phage particles. In addition, fast killing by the protein may lead to an increase in the phage-to-bacteria ratio (MOI). This would enhance the ability of the virus to exert a noticeable effect on the bacterial population. To test this possibility, the MOI in V329 biofilms treated with the phage-protein combination was compared to that in biofilms treated with the phage alone (Fig. 5). After 1 h of treatment, the starting MOI of 30.67 dropped to 11.89 and 0.70 in the phage and phage-protein treatment, respectively. However, this trend changed at later time points. Indeed, after 3 h of incubation, the MOI was consistently higher in the samples treated with phiIPLA-RODI combined with CHAPSH3b (Fig. 5). The MOI values after 3, 5, 7, and 24 h of treatment with phage alone were 1.42, 6.92, 4.78, and 0.46, respectively. In contrast, the values obtained at the same time points in the wells corresponding to the combination treatment were 137.55, 74.65, 510.80, and 43.48, respectively.

A similar trend was observed in *S. aureus* 15981, which forms strong, polysaccharide-based biofilms. In this strain, however, CHAPSH3b exerted a faster effect, with a significant reduction in cell numbers after just 1 h of incubation, and regrowth of protein-treated samples was slower than in V329 (Supplementary Fig. 1a). In this case, the MOI values were consistently higher in the samples treated with the protein-phage combination compared to those exposed only to the phage at all the analyzed time points. Indeed, the calculated MOIs after 1, 3, 5, 7, and 24 h of incubation were 0.001, 0.02, 0.10, 0.12, and 0.06 in samples treated with phiIPLA-RODI, while the values estimated for the combination treatment were 0.30, 16.85, 198.47, 46.70, and 33.94 (Supplementary Fig. 1b).

### CHAPSH3b can kill at least 90% of phage-resistant mutants

It is also a possibility that CHAPSH3b may limit phage-resistance development by killing resistant mutants. To better discern if this might be the case, ten BIMs of *S. aureus* V329 with resistance to phage phiIPLA-RODI were isolated to compare their CHAPSH3b susceptibility to that of the wild type. This was achieved by determining the specific lytic activity of CHAPSH3b against the different strains. The specific activity of the chimeric protein against the wild-type strain *S. aureus* V329 was 0.051 ΔOD_600nm_ × min^−1^ × mg protein^−1^, with similar results obtained for seven out of ten BIMs (BIM-2, BIM-4, BIM-6, BIM-7, BIM-8, BIM-9, and BIM-10) (Table 1). By contrast, the specific activity displayed by CHAPSH3b in mutants BIM-1, BIM-3, and BIM-5 was significantly lower (*p* < 0.05) compared to the wild type (Table 1). Nonetheless, it must be noted that in BIM-3 and BIM-5 the protein could still effectively eliminate bacterial cells, whereas BIM-1 exhibited resistance to CHAPSH3b. Of note, there was no significant difference in biofilm formation between the ten BIM strains and the wild-type strain (Supplementary Fig. 2).

**Table 1:** Specific lytic activity of protein CHAPSH3b against *S. aureus* V329 and *S. aureus* V329-derived BIMs.

Strains	Specific lytic activity^a^ ΔOD_600nm_ × min^−1^ × mg protein^−1^
V329	0.051 ± 0.004
BIM-1	−0.015 ± 0.010*
BIM-2	0.060 ± 0.023
BIM-3	0.033 ± 0.001*
BIM-4	0.039 ± 0.007
BIM-5	0.028 ± 0.009*
BIM-6	0.054 ± 0.013
BIM-7	0.067 ± 0.027
BIM-8	0.063 ± 0.040
BIM-9	0.073 ± 0.020
BIM-10	0.063 ± 0.011

^a^Values represent the means ± standard deviations from three independent replicates. *indicate values that were statistically different (*p* < 0.05) from those of the wild-type strain using an unpaired *t*-test with Welch’s correction.

### The combination of CHAPSH3b and phiIPLA-RODI limits bacterial regrowth in an ex vivo model of wound infection

**Fig. 6:**
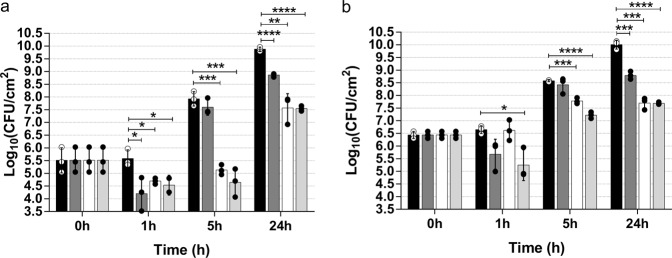
Antibiofilm effect of CHAPSH3b, phiIPLA-RODI or a combination of both on premature biofilms in an ex vivo model of intact and wounded skin. Antibiofilm effect of CHAPSH3b, phiIPLA-RODI or a combination of both on premature biofilms in an ex vivo model of intact and wounded skin. A porcine ex vivo model of intact skin (**a**) and skin wound (**b**) infection (*n* = 3) infected with 3-h-old biofilms of *S. aureus* V329 (~10^5^ CFU/g skin) was treated with either CHAPSH3b (8 µM), phiIPLA-RODI (MOI of 588) or a combination of both (8 µM and MOI of 588, respectively). Bars represent the means and standard deviations of three independent experiments. Bars with an asterisk are statistically different (*p* < 0.05) from the untreated control according to the Student’s *t*-test using the Holm-Sidak method.

To evaluate the antibiofilm potential of our compounds on a biotic surface, we implemented an ex vivo pig skin model of wound infection. Premature, 3-h-old biofilms established on both intact and wounded pig skin, were treated with 8 µM CHAPSH3b, phage phiIPLA-RODI (10^9^ PFU/ml, MOI = 588) or a mixture of both protein and phage (Fig. 6). Generally speaking, all treatments led to a significant reduction in the bacterial number after 1 h in intact skin. However, in wounded skin, this reduction was only significant for the combination treatment (*p* ≤ 0.05). Of note, treatments with CHAPSH3b and a combination of protein and phiIPLA-RODI resulted in a bacterial reduction of 1.3 and 1.04 log units, respectively, in the intact skin infection model (Fig. 6a). These values were almost the same as those observed in the wound infection model (1 and 1.4 log units, respectively, for the same treatments) (Fig. 6b). 5-h post-treatment, a significant difference in bacterial numbers was observed for the treatment with phiIPLA-RODI (0.8 log units) and the combination of CHAPSH3b/phiIPLA-RODI (3.3 log units). At the end of the experiment, all treatments resulted in a reduced number of viable bacteria compared to the untreated control. In both ex vivo models, the reduction in bacterial cells after 24 h of treatment was higher when using phiIPLA-RODI or the combination of phage and protein (~2.5 log units of reduction). In contrast, treatment with the protein alone led to final reductions of only ~1 log unit in both models.

## Discussion

Amidst the current antibiotic resistance crisis, bacterial biofilms pose a particularly dangerous threat. These complex multicellular structures are, by nature, considerably more resistant to antimicrobials than their planktonic counterparts. Moreover, when biofilms are formed by multidrug-resistant bacteria, the chances of successfully eliminating them are even lower. It is, therefore, necessary to find alternative strategies that can replace or complement the currently available antibiofilm agents. In this context, phage-based antimicrobials are promising candidates. Bacterial viruses themselves can be used for biofilm removal given their ability to specifically infect and kill their host, even when embedded in an extracellular matrix^48^. However, bacteriophage treatment is known to select phage-resistant bacteria, even though such resistant variants are often poor biofilm formers and/or display growth defects^39^. Phage-derived lytic proteins can also be powerful antibiofilm weapons that rapidly lyse their target cells without the significant selection of resistant variants^21^. Like phages, lytic proteins are quite specific and, as a result, harmless for non-target bacteria and, very importantly, eukaryotic cells. Moreover, some lytic proteins have been shown to kill persister cells, a characteristic that is a major asset for an antibiofilm agent^47^. Indeed, biofilms usually exhibit a higher proportion of persister cells than planktonic populations, a characteristic that boosts their ability to withstand an antimicrobial challenge^8^. On a critical note, lytic proteins do not increase in number during treatment and, depending on the environmental conditions, can often be unstable and only remain active for a relatively short time, thus allowing bacterial regrowth or requiring repeated dosing. This work explores the potential of harnessing the advantages of both phages and lytic proteins to compensate for each other’s weaknesses with the aim of developing a more effective antimicrobial combination.

Previous studies had already shown the ability of phage phiIPLA-RODI and chimeric protein CHAPSH3b to kill biofilm-embedded *S. aureus* cells belonging to certain strains^39,46^. As a result, they seemed a good choice to carry out interaction assays against three *S. aureus* strains. At the end of the 24-h treatment, neither antimicrobial was effective for controlling the bacterial population. In spite of this, exposure to the protein did decrease the amount of attached biomass in strains 15981 and V329, which might be due to its biofilm inhibiting properties^46^. Indeed, Fernández et al.^46^ already demonstrated that subinhibitory concentrations of this protein had a negative impact on biofilm formation by *S. aureus* that might be linked to downregulation of autolysin-encoding genes. The fact that this effect was not observed in strain IPLA1 might be a consequence of the weak biofilm formation of this strain in TSB without added glucose. In contrast to these results, a combination of both antimicrobials had a much more significant impact on the biofilm population for all three strains, independently of their susceptibility to phiIPLA-RODI. In view of these data, it appears that the application of phage phiIPLA-RODI combined with protein CHAPSH3b would constitute a viable antibiofilm strategy. Examples of combining these two phage-based strategies, i.e., phage therapy and lytic proteins, are scarce. In fact, we are only aware of one study in which a phage-endolysin combination was tested in a mouse sepsis model against *Acinetobacter baumannii*^49^. In this example, the combination gave similar results to the endolysin alone, although it is worth mentioning that the authors only tested one concentration of phage and protein against a single bacterial strain. With that in mind, it cannot be excluded that different conditions might have led to the observation of a synergistic interaction. Nonetheless, it is likely that, as is the case of other antimicrobials, the existence of synergy will depend on the specific phage-protein combination and the bacterial strain. There are several studies reporting the existence of synergy between either phages or phage lytic proteins with antibiotics^36–38,41–43^. In some cases, such combinations were able to reduce the biofilm population depending on the antibiotic and its concentration. Nevertheless, there is evidence that antibiotics can also have a negative impact on phage treatment. This is particularly the case for drugs that inhibit nucleic acid or protein synthesis, as they collaterally interfere with phage propagation^37^. However, this antagonism depends on the duration of the treatment and the antibiotic concentration used^37,50^. Moreover, any strategy involving conventional antimicrobials has the potential risk of contributing to the antibiotic resistance problem. These two important shortcomings of phage/antibiotics combination therapy would be avoided when combining phages and lytic proteins.

Once ascertained that combining a virulent phage, phiIPLA-RODI, with CHAPSH3b for biofilm treatment has a positive impact, we sought to better understand the basis for this interaction. The results of this analysis suggest that the presence of the phage, which has no effect by itself, appears to limit the regrowth of the bacterial population that follows the inactivation of the lytic protein. On the other hand, the protein, by lysing part of the bacterial population, increases the phage-to-bacteria ratio, thereby allowing the phage to exert a significant impact on biofilm removal. In addition to changing the phage-to-bacteria ratio, CHAPSH3b might also enhance phage efficacy by killing potential phage-resistant variants present in the biofilm. Indeed, our results show that at least 90% of BIMs are susceptible to the lytic protein. Finally, a third potential contribution of the lytic protein might be by loosening the biofilm, thus making cells more accessible to phage infection.

When comparing the different antibiofilm strategies in ex vivo experiments using the pig skin model, we observed that phiIPLA-RODI was more effective than CHAPSH3b in limiting the growth of the bacterial population in the long term. This might be associated with the fact that porcine skin is a nutrient-rich environment that favors bacterial growth and, consequently, facilitates phage propagation. Nonetheless, it must be noted that the combination treatment generally led to a faster decrease in the short term compared to the phage alone.

This study shows how we can take advantage of the synergistic interaction between bacteriophages and lytic proteins to develop a two-speed antibiofilm cocktail. In this model, the lytic proteins initiate rapid killing of the microbial community. Once the lysin activity dwindles, the phage present in the mixture will continue its antimicrobial action. This slower but longer-term effect limits regrowth of the target bacterium, thereby facilitating removal by subsequent treatment. Overall, this strategy provides an interesting antibiotic-free alternative for biofilm elimination with a low potential for resistance selection.

## Methods

### Bacterial strains, lytic proteins, bacteriophages, and growth conditions

*S. aureus* strains used in this study included the dairy industry isolate *S. aureus* IPLA1^51^, the clinical strain *S. aureus* 15981^52^ and the bovine subclinical mastitis isolate *S. aureus* V329^15^. These bacterial strains were routinely grown at 37 °C in TSB (tryptic soy broth, Scharlau Microbiology, Barcelona, Spain) by shaking or on plates containing TSB supplemented with 2% (w/v) agar (Roko, S.A., Llanera, Spain) (TSA). TSB top agar composed of TSB supplemented with 0.7% (w/v) agar was used for phage titration. TSB supplemented with 0.25% (v/v) glucose (Merck, Darmstadt, Germany) (TSBg) was used for biofilm formation assays.

Recombinant protein expression was carried out using *Escherichia coli* BL21 carrying the gene coding for CHAPSH3b cloned into plasmid pET21a as described by^35^. *E. coli* was routinely grown in LB medium, supplemented with 1 mM IPTG and 100 μg/ml ampicillin when necessary. The chimeric protein CHAPSH3b was subsequently purified as described previously^35^. Visual analysis and quantification of the concentration of the purified protein were performed by SDS-PAGE and the quick Start Bradford Protein Assay Kit (Bio-Rad Laboratories, USA), respectively.

Phage phiIPLA-RODI was routinely propagated on *S. aureus* IPLA16 and partially purified by adding 10% polyethylene glycol (PEG) and 0.5 M NaCl for incubation at 4 °C for 16 h. Concentrated phage was obtained by centrifugation (10,000 rpm, 30 min, 4 °C), resuspended in TSB medium, and stored at 4 °C until further use.

### EOP determination and phage adsorption assays

To determine the EOP of phage phiIPLA-RODI on the different strains, the phage titer on the test strain was divided by the titer on strain *S. aureus* IPLA1.

To estimate the phage adsorption rate, overnight cultures of the different *S. aureus* strains were diluted to an OD_600_ of 1. Next, 900 μl aliquots from these suspensions (~10^8^ CFU/ml) were mixed with 100 μl of a phiIPLA-RODI stock leading to a final concentration of 10^7^ PFU/ml (MOI = 0.1). The negative control was prepared by combining 900 μl of non-inoculated TSB with 100 μl of the phage stock. Phage adsorption was then allowed to occur for 5 min at room temperature. The samples were subsequently centrifuged for 3 min at 10,000×*g* at 4 °C. The number of non-adsorbed phages was calculated by titrating the resulting supernatants, and the phage adsorption rate was determined according to the following equation:

1 $$\begin{array}{l}{\mathrm{Phage}}\,{\mathrm{adsorption}}\,{\mathrm{rate}} = \left[\left( {\mathrm{phage}}\,{\mathrm{number}}\,{\mathrm{in}}\,{\mathrm{supernatant}}\,{\mathrm{of}}\,{\mathrm{control}}\right.\right.\\\left.\left.-\,{\mathrm{phage}}\,{\mathrm{number}}\,{\mathrm{in}}\,{\mathrm{supernatant}}\,{\mathrm{sample}}\right)/ \left( {{\mathrm{phage}}\,{\mathrm{number}}\,{\mathrm{in}}\,{\mathrm{supernatant}}\,{\mathrm{of}}\,{\mathrm{control}}} \right)\right]\\ \times\,100\end{array}$$

### Biofilm formation and treatment

Overnight cultures of each *S. aureus* strain were diluted 1:100 (v/v) in fresh TSBg medium. Then, 1 ml of this bacterial suspension was inoculated into each well of a 24-well polystyrene microtiter plate (Thermo Scientific, Nunclon^TM^ Delta Surface) and the plates were incubated for 24 h at 37 °C. Afterward, the planktonic phase was removed, and the biofilms were washed twice with phosphate-buffered saline (PBS; 137 mM NaCl, 2.7 mM KCl, 10 mM Na_2_HPO_4_, 2 mM KH_2_PO_4_ [pH 7.4]). The remaining adhered cells were then treated with 0.5 ml of TSB medium alone or using the same medium with different concentrations of protein CHAPSH3b (4–8 µM) and/or phage phiIPLA-RODI (1 × 10^9^ PFU/ml–1 × 10^10^ PFU/ml) at 37 °C. Treatment was allowed to act for 1, 3, 5, 7, or 24 h. Then, the planktonic phase was removed and the adhered phase was washed twice with PBS. To assess the efficacy of the different treatments, the number of viable attached cells and total biomass were quantified. The number of viable cells present in the biofilms was determined by using the spot test. Briefly, biofilms were scraped and resuspended in PBS. Afterward, 10 µl droplets from tenfold serial dilutions of this cell suspension were spotted onto TSA plates and allowed to dry. These plates were then incubated at 37 °C for 24 h.

The cell counts obtained in these experiments were first used to determine the number of CFUs per unit area (CFU/cm^2^) and, subsequently, the potential interaction between the two antimicrobials (phage and lysin) as indicated using the following equation^53^:

2 $$\begin{array}{l}\left[ {{\mathrm{log}}_{10}{\mathrm{CFU}}/{\mathrm{cm}}^2\left( {{\mathrm{phage}} + {\mathrm{lysin}}} \right)} \right] - \\ \left[ {{\mathrm{log}}_{10}{\mathrm{CFU}}/{\mathrm{cm}}^2\left( {{\mathrm{phage}}} \right) + {\mathrm{log}}_{10}{\mathrm{CFU}}/{\mathrm{cm}}^2\left( {{\mathrm{lysin}}} \right)} \right]\end{array}$$

The values obtained with the aforementioned equation were named interaction indices. The interaction was considered additive when this index was between −0.5 and 0.5, antagonistic when the value was <−0.5, and synergistic when the value was >0.5.

Total biomass was quantified by performing the crystal violet staining assay as described previously^47^. Briefly, after washing the biofilm with PBS, 1 ml of 0.1% (w/v) crystal violet was added to each well. Fifteen minutes later, the excess of crystal violet was removed by washing twice with water. The remaining dye was then solubilized by adding 33% (v/v) acetic acid and the absorbance at 595 nm (A_595_) was measured using a Benchmark Plus Microplate Spectrophotometer (Bio-Rad Laboratories, Hercules, CA, USA).

To monitor the evolution of the MOI throughout treatment, the number of viable cells and phage particles was determined in both the planktonic phase and the biofilm. Next, the values obtained for both phases were added to calculate the total number of infective phage particles and bacterial cells, and the ratio between the two populations was calculated to determine the MOI by dividing the number of PFUs by the number of CFUs.

### Determination of minimum inhibitory concentrations (MICs)

The MICs of the protein and the phage were determined using the broth microdilution technique in TSB medium with some modifications^54^. Thus, in the case of the phage, tenfold dilutions of the viral suspension were assayed instead of the usual twofold dilutions. The MIC was defined as the lowest concentration that inhibited visible bacterial growth after 24 h of incubation at 37 °C. The experiment was performed in triplicate and the MIC was expressed as the mode of three independent replicates.

### Isolation of bacteriophage insensitive mutants (BIMs)

BIMs of *S. aureus* V329 were isolated as described previously^39^. Briefly, 100 µl from an overnight culture were mixed with 100 µl of phage (5 × 10^9^ PFU/ml), spotted onto the center of a 2% TSA plate and covered with 0.7% TSA. Afterward, the plates were incubated for 24 h at 37 °C. Some of the surviving colonies were picked with a pipette tip and grown in fresh TSB medium for 16 h at 37 °C. The insensitive phenotype of the selected colonies was then confirmed by using the spot assay.

### Quantification of the CHAPSH3b specific lytic activity

Overnight cultures of *S. aureus* V329 and the ten isolated BIM strains were grown at 37 °C with shaking and then the turbidity reduction assay was performed as described previously with some modifications^55^. Briefly, after reaching an OD_600_ of 0.5–0.6, the bacterial cells were washed and then resuspended in NaPi buffer (50 mM, pH 7.4) at a final OD_600_ ~1.0. Next, the freshly prepared cell suspensions were treated with two-fold dilutions of purified CHAPSH3b (0.027–1.720 mg/ml). The specific lytic activity of the protein was expressed in OD_600nm_ × min^−1^ × mg protein^−1^ ^56^.

### Analysis by CLSM and time-lapse microscopy

Confocal and time-lapse microscopy were performed as described previously, with some modifications^46^. For confocal microscopy analysis, 24-h-old biofilms were formed by inoculating 2 ml of an *S. aureus* V329 cell suspension containing approximately 10^6^ CFU/ml in TSBg in two-well µ-slides with a glass-bottom (ibidi, Martinsried, Germany), and subsequent incubation under static conditions at 37 °C. After growth, the planktonic phase was removed and the biofilm was washed twice with PBS. Then, TSB medium alone or containing phage phiIPLA-RODI (5 × 10^9^ PFU/ml), protein CHAPSH3b (8 µM), or both together were added to the biofilm and incubated for 24 h at 37 °C. At the end of the treatment, wells were washed twice with PBS and stained with the Live/Dead® BacLight^TM^ kit (Invitrogen AG, Basel, Switzerland). Samples were observed under a confocal scanning laser microscope (DMi8, Leica Microsystems) using a 63 × oil objective.

For time-lapse microscopy, 24-h-old biofilms were grown at 37 °C and then washed twice with PBS. Next, different treatments were added to the biofilm: TSB containing protein CHAPSH3b (8 µM) or phage phiIPLA-RODI (5 × 10^9^ PFU/ml) combined with protein CHAPSH3b. Then the plate was placed in an incubation chamber, set at 37 °C, and connected to an inverted microscope (DMi8; Leica Microsystems) equipped with a Leica DFC365FX digital camera. Images were acquired every 15 min using LasX software (Leica Microsystems) for approximately 24 h.

### Ex vivo pig skin model of wound infection and treatment

To assess the antibiofilm effect of CHAPSH3b and phiIPLA-RODI on a biotic surface, a previously described pig skin model was used with minor adaptions^57^. Pig skin was obtained from the Minimally Invasive Surgery Center Jesús Usón (Cáceres, Spain). First, the upper layer was disinfected with 70% ethanol. This disinfection was repeated after removing residual hair. Next, the skin was cut in 1 × 1 cm explants. To mimic a wound phenotype, a wound bed of 48 mm diameter and 1 mm depth was made using a hand drill with a cutter bit (Dremel® #192) in specific explants. Next, all the explants, with or without a wound, were submerged in 70% ethanol for 1 h followed by 30 min of UV decontamination to ensure complete sterility. Two different experimental setups were carried out in parallel, for explants with and without a wound. For each time point, three explants without a wound and three explants with a wound were placed in 24-well plates containing 1 ml physiological saline agar (0.9% (w/v) NaCl, 0.5% (w/v) agar, pH 5.5) to mimic human skin conditions. The explants were inoculated with *S. aureus* V329 (10^5^ CFU/g of skin) and incubated at 37 °C (5% CO_2_) for 3 h to allow for biofilm formation. Then, the explants were treated with 100 µl CHAPSH3b (8 µM), phiIPLA-RODI (MOI = 588), or 100 µl of a mixture containing CHAPSH3b (8 µM) and phiIPLA-RODI (MOI = 588). Treatment with 100 µl of TSB was used as a negative control. Explants were processed after 0, 1, 5, and 24-h of treatment (37 °C, 5% CO_2_). After incubation, bacteria were recovered by inserting the explant in a stomacher bag (BagPage, BagSystem, Interscience, St-Nom-la-Breteche, France) containing 5 ml of PBS and homogenized using a stomacher for 2 × 90 s (model 80, Seward Medical, London, UK). From this, a tenfold dilution series was made in PBS, and colony-forming units (CFUs) were determined by plating duplicates on Baird-Parker agar plates that were incubated for 16-h at 37 °C.

### Statistical analysis

Statistical analysis of the biofilm data was carried out by multiple *t*-tests, using the Holm-Sidak method or Welch’s correction using GraphPad Prism 6 software. *P*-values lower than 0.05 were considered significant.

### Reporting summary

Further information on research design is available in the Nature Research Reporting Summary linked to this article.

## Supplementary information

Supplementary Movie 1

Supplementary Movie 2

Supplementary Information

Reporting Summary

## Data Availability

All relevant data used to support the findings of this study are included within the article. Additional information and data are available from the authors upon reasonable request.
