# Supplementary material for: Synergistic action of phage phiIPLA-RODI and lytic protein CHAPSH3b: a combination strategy to target Staphylococcus aureus biofilms
Source: NPJ Biofilms Microbiomes. 2021 Apr 22;7:39. doi: 10.1038/s41522-021-00208-5 (PMC8062563; doi:10.1038/s41522-021-00208-5)
Supplement: Supplementary file 3 — Supplementary Information [file 41522_2021_208_MOESM3_ESM.pdf]

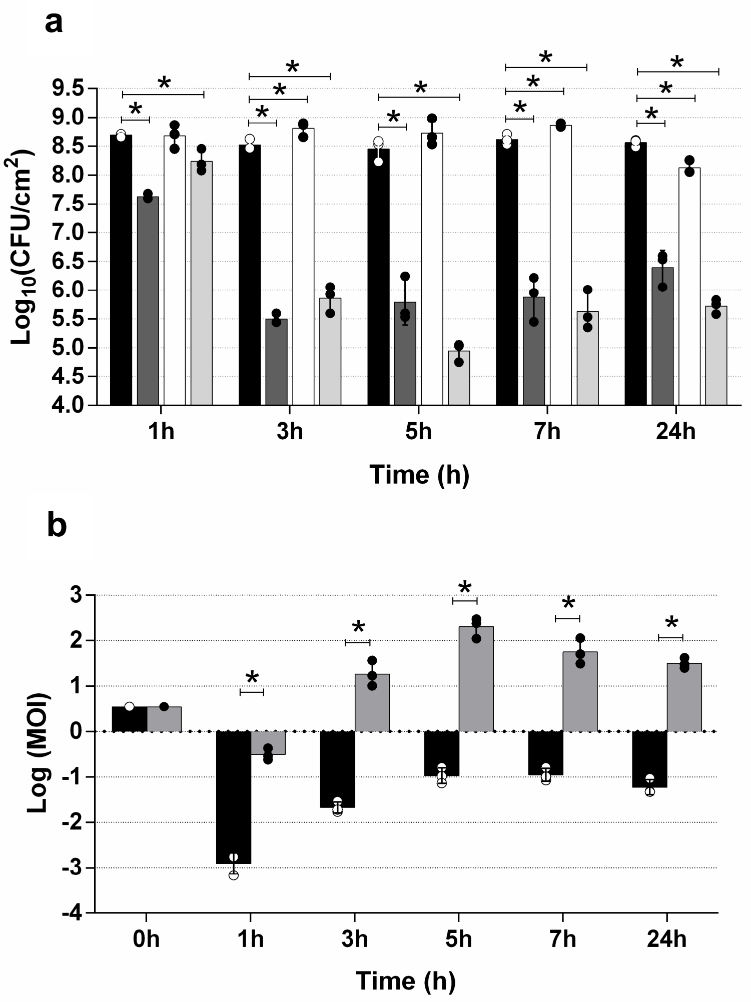

Supplementary Figure 1. Time-kill curve of *S. aureus* 15981 biofilms treated with protein CHAPSH3b and/or phage phiIPLA-RODI. a) 24-h-old biofilms were treated with protein at 8 μM (grey bars), phage at 1×10^9 PFU/ml (white bars) or a combination of both (light grey bars) and incubated for 1, 3, 5, 7 or 24 hours at 37ºC. Control wells were treated with TSB medium alone (black bars). Data correspond to the means ± standard deviations of three independent experiments, and represented in logarithmic scale in colony forming units per cm^2 of biofilm. Bars with an asterisk are statistically different ($p<0.05$) from the untreated control according to the Student's t-test using the Holm-Sidak method. b) 24-h-old biofilms were treated with phage at 1×10^9 PFU/ml (black bars) or a combination of phage at 1×10^9 PFU/ml and protein at 8 μM (grey bars). Data correspond to the means ± standard deviations of three independent experiments, and represent the logarithm of the MOI for each time point. Bars with an asterisk are statistically different ($p<0.05$) from each other according to the Student's t-test using the Holm-Sidak method.

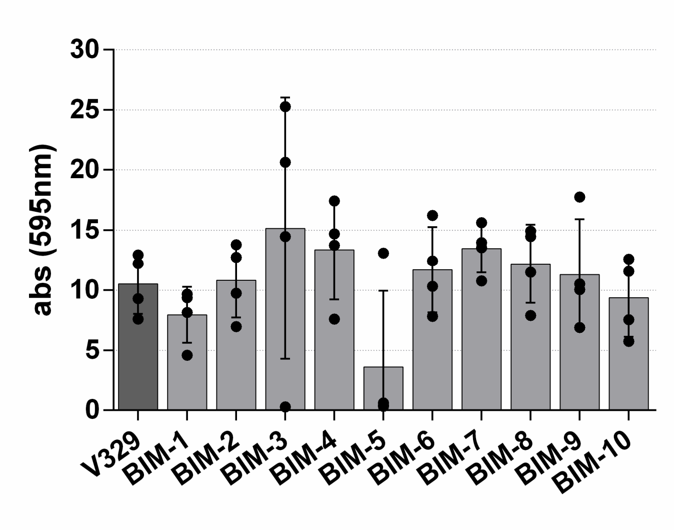

Supplementary Figure 2. Biofilm formation of BIMs derived from strain V329 after 24 hours of incubation at 37ºC. The depicted values correspond to the average and standard deviation of three independent repeats. After growth, biofilms were stained with crystal violet and A_{595} was then measured to quantify attached biomass. * P-values < 0.05 were considered significant according to the Student's t-test using the Holm-Sidak method

Supplementary Video 1. Time-lapse microscopy analysis of 24-hour biofilms treated with lytic protein CHAPSH3b (8 µM).

Supplementary Video 2. Time-lapse microscopy analysis of 24-hour biofilms treated with lytic protein CHAPSH3b (8 µM) and phage phiIPLA-RODI (1 × 10^9 PFU/ml).
